# Learning analytics in support of inclusiveness and disabled students: a systematic review

**DOI:** 10.1007/s12528-023-09363-4

**Published:** 2023-03-14

**Authors:** Mohammad Khalil, Sharon Slade, Paul Prinsloo

**Affiliations:** 1 Centre for the Science of Learning & Technology (SLATE), University of Bergen, Christiesgate 12, Bergen, 5020 Norway; 2 EarthTrust, Little Wittenham, Abingdon, OX14 4QZ UK; 3 University of South Africa, P O Box 392, Unisa, Pretoria, 0003 South Africa

**Keywords:** Learning analytics, Inclusiveness, Disability, Disadvantaged groups, Disadvantaged students

## Abstract

This article maps considerations of inclusiveness and support for students with disabilities by reviewing articles within the field of learning analytics. The study involved a PRISMA-informed systematic review of two popular digital libraries, namely Clarivate’s Web of Science, and Elsevier’s Scopus for peer-reviewed journal articles and conference proceedings. A final corpus of 26 articles was analysed. Findings show that although the field of learning analytics emerged in 2011, none of the studies identified here covered topics of inclusiveness in education before the year of 2016. Screening also shows that learning analytics provides great potential to promote inclusiveness in terms of reducing discrimination, increasing retention among disadvantaged students, and validating particular learning designs for marginalised groups. Gaps in this potential are also identified. The article aims to provide valuable insight into what is known about learning analytics and inclusiveness and contribute knowledge to this particular nascent area for researchers and institutional stakeholders.

## Introduction

It is often assumed in education that benefits are equally distributed, that everyone has equal chances of succeeding and that the educational ecosystem is, per se, value and power-free. One of the dominant beliefs is that all learners can succeed if they try hard enough, show grit and resilience and take control of their learning and opportunities (e.g., Reed & Jeremiah, 2017; Warren & Hale, 2020). However, grit and resilience may look very different in the context of learners from marginalised groups, students with disabilities and students studying in their second or third language. If we view education as an ecosystem, it necessitates a critical evaluation of how the benefits between actors are shared, and various values and powers inform that ecosystem.

Uses of digital learning platforms within educational settings are becoming the norm. While classroom-based teaching remains a mainstay of teaching and learning, it is perhaps time to examine the broader impact of newer digital approaches. Though student data has always been used in education, the increased volumes, variety, and velocity of data led to a new research focus and practice called learning analytics (Long & Siemens, 2011). The definition of learning analytics was established in 2011 and is generally accepted to be the measurement, collection, analysis and reporting of data about learners and their contexts, for purposes of understanding and optimising learning and the environments in which it occurs (Long & Siemens, 2011).

As learning is transitioning towards being digital and datafied, student data from digital learning environments can provide stakeholders (e.g., students, instructors, administrators, student centres, etc.) with actionable information (Khalil et al., 2018). That is, it has the potential to facilitate the design and implementation of more appropriate and effective learning pedagogies, empower active learning, identify factors impacting student success, and support the design of courses to meet students’ individual needs (Samuelsen et al., 2019; Nguyen, Tuunanen, Gardner & Sheridan, 2021). With its associated methods in data collection, analysis and machine learning, learning analytics therefore has great potential to address critical issues related to student engagement, success, progression, and retention (Khalil & Ebner, 2015). The latter potential has been an area of particular focus for learning analytics, and research on this topic draws on large-scale studies that have sought to explain student attrition behaviours in higher education (HE) settings (Prinsloo & Slade, 2017).

Despite some debate around whether the field has matured and significantly evolved, the initial definition holds firm^1^. With thousands of papers published in the domain (Khalil et al., 2022), the field of learning analytics retains its potential to positively influence educational outcomes. Although learning analytics is able to make pragmatic and theoretical contributions to optimise and support learning, obstacles remain hindering its growth toward effective, scalable, ethical and measurable impacts. Selwyn (2019) listed a number of these issues as: (1) A reduced understanding of education; (2) Ignoring the social context of education; (3) A reduction in student and teacher capacity for informed decision-making; (4) Learning analytics designed for surveillance rather than support; (5) Institutions as the main beneficiaries (rather than students); and (6) Large groups of students being (dis)advantaged. The final point touches on the theme of our article in that learning analytics may benefit some student groups more than others.

Learners, arguably the main stakeholders for learning analytics, are not *all* equal. For instance, it has been noted that students with undeclared disabilities (e.g., asthma and diabetes) do not perform as well as those without disabilities (Ferguson, 2019). Additionally, studies report that students with disabilities have lower completion rates than those without (Cooper et al., 2016; Ferguson, 2019). The reasons for this will vary, but may relate to a lack of appropriate interventions and suitable accommodation for particular disabilities.

Of the large body of learning analytics which researches inclusivity and students with disabilities, few have investigated how learning analytics can better serve underrepresented groups of students such as students with disabilities, students from different minorities, or those who are socially disadvantaged in both contexts of higher education and schools. This study is motivated by the frequent calls for learning analytics to promote inclusivity and support for students with disabilities (Uttamchandani & Quick, 2022; Williamson & Kizilcec, 2022). Its aim is to help inform institutions and the research community by describing what has been researched and already considered. To that end, we follow a systematic literature review to answer the following research question:

*What is known about learning analytics in promoting inclusiveness and supporting students with disabilities?*

The review study is structured as follows: A relevant background is first established followed by a narrative reporting on a systematic review of the literature. We then share the findings of our synthesis and results and discuss key insights and findings. Finally, limitations are explored, and conclusions drawn.

## Background

Educational Inclusiveness is part of the human rights of social inclusion (Vrooman & Coenders, 2020). It is important, because inclusive education supports disadvantaged and marginalised groups of people in taking part in the community and in securing a gateway to improved well-being via education. While inclusive education covers a variety of underrepresented individuals, those with disabilities are the majority. The United Nations (UN) defines persons with disabilities as “those who have long-term physical, mental, intellectual or sensory impairments in which interaction with various barriers may hinder their full and effective participation in society on an equal basis with others.” The UN Convention on the Rights of Persons with Disabilities (UN, 2015) furthermore vouchsafes the full scope of human rights in all areas of society, including the right to academic inclusion and lifelong learning, and UNESCO promotes the establishment of equal, fair, and open educational environments and opportunities (UNESCO, 1994). This position is acknowledged in the 2030 Agenda for Sustainable Development, which emphasises the importance of ensuring equal access to all levels of education and vocational training for persons with disabilities by 2030 (UN, 2015).

There is a moral obligation then to realise and improve research-informed access to quality education for individuals living with disabilities and for other minority groups. Given evidence that the number of students with disabilities within higher education is continuing to increase (Seale et al., 2015; Moriña, 2017), and also in schools (Kourakli et al., 2017), many countries have begun to focus effort on supporting access for people with disabilities by increasing inclusivity and dismantling discrimination. Though such efforts should be applauded and sustained (UN, 2015), the work in this direction remains limited (Chen, 2020).

In times of crises, such as pandemics, institutional support for marginalised and students with disabilities may be overlooked as educational institutions move to emergency remote teaching and learning. With the increasing move to online learning (whether in response to international trends or crises), the mainstreaming and integration of support for those with disabilities in the use of educational technologies may have become less of a priority. (Un)intentional exclusion of students who are disadvantaged may further drive them into more vulnerable subgroups of society where educational needs are neglected and there is a lack of attention and/or allocation of resources needed (Berger et al., 2020). Even before COVID-19, and as reported by Lombardi, Murray, and Kowitt (2016), disadvantaged students in higher education were at ‘greater risk’ of prematurely withdrawing or dropping out from universities compared to students without disabilities.

In discussing the potential of learning analytics to promote inclusivity and, more specifically, for those student with disabilities, Chen (2020) suggests that the prospects for institutions to apply learning analytics to better support students with disability could be significant, e.g., by informing design support systems (e.g., enabling captions for hearing impaired students based on data analytics, providing automatic text adjustment for students with poor vision, etc.).

## Methodology

**Fig. 1:**
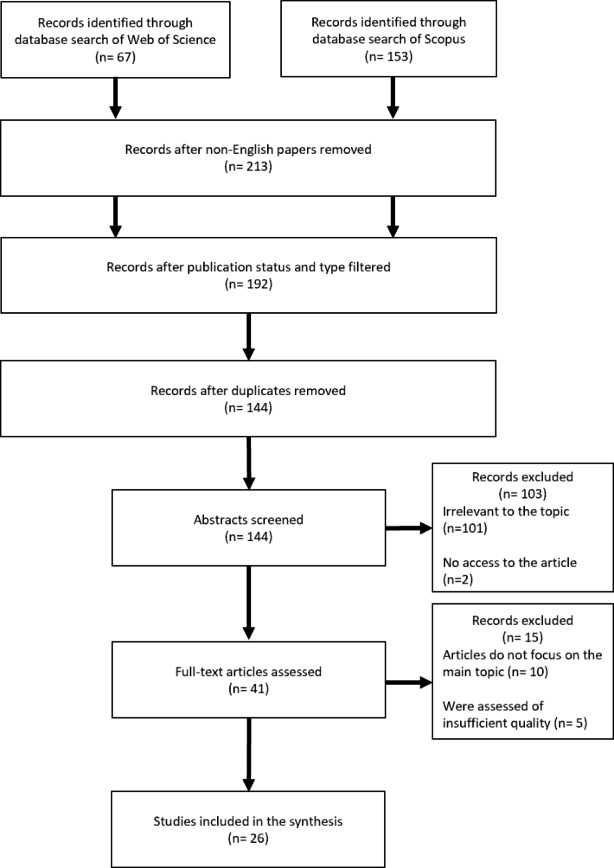
PRISMA flow chart in the systematic review, as adopted by Page et al. (2021)

The research focus of this systematic review is to better understand the literature on the growing area of learning analytics as related to inclusiveness and disabilities. As Alexander (2020) proposes, systematic reviews are based around a well-defined research question in an attempt to address areas of scarce knowledge. As such, she stresses that systematic reviews can provide new perspectives to educational research as compared to other research methodologies. Since this study focuses on learning analytics as a driver to our topic of interest (i.e., inclusiveness), we opted to adopt a systematic review to address the research question. As shown in Fig. 1, our approach adopts the checklist and guidelines of Preferred Reporting Items for Systematic Reviews and meta-analyses (PRISMA) developed by (Page, McKenzie, Bossuyt, Boutron, Hoffmann, Mulrow, … & Moher, 2021) to ground transparency in the process of library searches, filtration, and analysis for later collation and synthesis.

The PRISMA procedure (see Fig. 1) involved the following stages: (1) a search of two digital libraries: Clarivate’s Web of Science and Elsevier’s Scopus; (2) removal of results according to specific exclusion criteria; (3) removal of duplicates; (4) initial scanning of paper abstracts and exclusion of those deemed not relevant; (5) careful collation of the remaining full articles and exclusion of any not fulfilling the selection criteria; and (6) deeper synthesis of each article to review and extract relevant content and contribution in support of addressing the research question. Throughout this approach, two of the three authors met regularly to ensure consistency of the processes and transparency in terms of interrelated reliability.

### Data search strategy

The systematic review focused on established database providers (i.e., Scopus and Web of Science) and avoided grey literature crawler-based search engines as advised by the recent publication “Which academic search systems are suitable for systematic reviews or meta-analyses? Evaluating retrieval qualities of Google Scholar, PubMed, and 26 other resources” (Gusenbauer & Haddaway, 2020). The keywords searched for were bigrams of “learning analytics” and truncations of “inclusiveness”, “disability”, and “disadvantaged” in the title, abstract, and author keywords as follows:

- Web of Science.
  - TS=(“Learning Analytics”) AND (TS=(inclus*) OR TS=(disab*) OR TS=(disadvant*)).
- Scopus.
  - (TITLE-ABS-KEY ( “Learning Analytics” ) AND ( TITLE-ABS-KEY ( “inclus*” ) ) OR ( TITLE-ABS-KEY ( “disab*” ) ) OR ( TITLE-ABS-KEY ( “disadvant*” )))

The timeline of the search begins with the formal emergence of the field of learning analytics in January 2011 till the date of the search in April, 2022.

### Inclusion and exclusion criteria

A preliminary search of the two digital databases yielded more than 350 possible papers, articles, and reports. However, we limited the search to peer-reviewed journal articles and conference proceedings. Books, book chapters, workshop papers, posters, dissertation, reports and editorials were excluded, yielding 220 journal articles and conference proceedings papers. As advised in PRISMA, one of the major steps is to define clear and precise inclusion and exclusion criteria when conducting a systematic review. Table 1 describes in detail the criteria applied in this work.

**Table 1:** Inclusion and exclusion criteria followed in the systematic review

Criteria	Inclusion	Exclusion
Topic and focus	Research work of learning analytics on inclusiveness, disabilities, and disadvantaged groups	Research papers that are not specifically themed on learning analytics for inclusiveness, disabilities and disadvantaged groups of students
Publication status	Peer-reviewed journals and conference and published papers	Non peer-reviewed and Articles In Press (AIP)
Publication type	Journal articles and conference papers	Dissertations, books, book chapters, workshop papers, posters, editorials and reports
Publication date	Jan, 2011- April, 2022	Outside the particular time frame
Language	English	Other languages

### Coding scheme

The coding for each of the included papers is listed in Table 2. The authors coded the final corpus of articles by summarising information to include the paper title, authors, year, context, aspects of inclusiveness, methodology, the purpose of learning analytics, and whether the evaluated study had been evidenced.

**Table 2:** Coding scheme used to describe the included papers

Coding scheme	Description
Paper title	Title of the included paper
Authors (year)	Authors of the paper with the year of publication
Context	Pre Higher Education (Pre HE), Higher Education (HE), non-specific, adult education, and special education
Description of Inclusiveness	What aspect(s) of inclusiveness, disability, or (category of) disadvantaged group is addressed?
Methodology	What methodology is adopted? e.g., case study, literature review, pilot study, technical overview, …etc.
Purpose of learning analytics	What is learning analytics seeking to address (e.g. retention, personalisation, support, etc.)?
Evidenced	Has the study been evidenced? e.g., through empirical findings, surveys, interviews, framework development.etc.

### Reliability

To address issues of bias in this study, we calculated inter-rater reliability (IRR). IRR refers to the consistency of resolution between authors in the context of academia. It has become a recommended element to construct validity of studies, particularly for systematic reviews (Cook & Beckman, 2006). In this study, we used Fleiss kappa as a measure to evaluate the inter-rater agreement among the authors. Fleiss, Levin, and Paik (2013) propose that Fleiss kappa values of over 0.81 describe a very good level of agreement between authors, 0.61 ~ 0.80 describe a good agreement, 0.41 ~ 0.60 indicates a fair consensus, and values below 0.41 indicate a poor level of agreement between authors.

In this case, two of the authors scanned the abstracts of all filtered papers and identified papers for inclusion and exclusion for further detailed scrutiny. Discussions were held regarding areas of uncertainty and agreement reached. To calculate the IRR value, we adopted an R software package called ‘irr’ developed by Gamer, Lemon, Gamer, Robinson, & Kendall’s (2012) and uploaded a three-valued logic (agree, disagree, natural) comma-separated file that coded agreement and disagreement throughout the scanned articles. The final IRR kappa when comparing the results of the two authors indicated a good level of agreement (κ = 0.73, subjects = 144, raters = 2, and p < 0.005) with low measure of controversy.

## Findings

### Prisma results

The total number of results appearing from the search in the two digital libraries returned 213 papers. Further filtration of the publication status excluded those in press, or non-peer reviewed (n = 21). Next, we removed duplicates (n = 48). Screening as described above in the methodology section further excluded (n = 103) articles which were either inaccessible (n = 2) or deemed irrelevant (n = 101). The final process of exclusion removed articles that were out of the focus scope (n = 10) or were assessed of insufficient quality (n = 5). The final corpus included 26 journal articles and conference proceedings.

### Overview of the results of the final corpus

As a basis for answering our research questions, Table 3 shows the result of coding the final list of articles. The paper authors and year of publication are noted in the first column. The second column denotes the context of the studies, i.e., higher education, pre-higher education, non-specific/generic, special education, and elderly/adult education. The third column describes the aspects of inclusiveness covered in the reviewed studies. The last three columns denote the particular methodology followed, purpose of learning analytics in shielding the aspect of inclusiveness, and whether it has been empirically evidenced. Further details of the papers are later discussed.

**Table 3:** An overview of articles included in the final analysis

Authors (year)	Context	Description of Inclusiveness	Methodology	Purpose of Learning Analytics	Evidenced
Williamson & Kizilcec (2022)	Higher Education	Socio-demographic achievement gaps; under representation of historically disadvantaged groups	Critical literature review	State the field; improve application of learning analytics dashboards; support of inclusiveness, equity, and diversity	No
Mohammadhassan & Mitrovic (2021)	Higher Education	English as a foreign language (EFL)	Surveys and case studies	Video based learning system which automatically assess the quality of comments written by students and guide them toward critical thinking and self-reflection. Study aims to note and address improvements which take account of EFL students and improve learning outcomes	Yes
Dietrich, Greiner, Weber-Liel, Berweger, Kämpfe, & Kracke (2021)	Higher Education	Learning Disability	Randomised control trial	Differentiated instruction for formative self-assessment by offering computerized feedback	Yes
Bayer, Hlosta, & Fernandez (2021)	Higher Education	BAME (black, asian and minority thnic), Gender and disability	Case study	Predictive analytics with support interventions - retention	Yes
Summers, Higson, Moores (2021)	Higher Education	Ethnic minorities; poor students	Case study	Monitoring behaviour	Yes
Costas-Jauregui, Oyelere, Caussin-Torrez, Barros-Gavilanes, Agbo, Toivonen, Motz, Tenesaca (2021)	Higher Education and Pre Higher Education	Learning disability	Prototype, teacher interviews	Learning Analytics dashboards - inclusive of students with disabilities	Yes
Hlosta, Herodotou, Bayer, & Fernandez (2021)	Higher Education	BAME; low socio-economic status groups	Case study	Predictive analytics with support interventions - outcomes	Yes
Tsikinas &Xinogalos (2021)	Special education	Learning disability; autism spectrum disorder	Prototype; case study	Game-based approach to support development of life skills	Yes
Chen (2020)	Higher Education	Disability (general)	Position paper	Non-specific	No
Niemelä, Kärkkäinen, Äyrämö, Ronimus, Richardson, & Lyytinen (2020)	Pre Higher Education	Learning disability	Case study	Serious games analytics; profiling reading disability	Yes
Foster & Siddle (2020)	Higher Education	Social disadvantage	Position paper	Designing a dashboard; support; retention	No
Selwyn (2019)	Higher Education	Disability (general); minorities	Position paper	Non-specific	No
Oyelere, Silveira, … Tomczyk, (2020)	Higher Education	Non-specific; inclusion	Design research	Learning Analytics as a small part of a larger digital ecosystem; progress tracking; predictive analytics - support and learning interventions	No
Alonso-Fernández et al., (2019)	Non-specific	Learning disability	Case Studies; Design research, Surveys and Questionnaires	Case Studies; Design Experiments; Surveys and Questionnaires	Yes
Riazy & Simbeck (2019)	Higher Education	Gender; disability	Case study	Predictive analytics	Yes
Tamura et al. (2019)	Adult education	Age	Case studies and prototype development	Learning Analytics to support learning problems via multimodality	Yes
Nguyen, Gardner, Sheridan (2018)	Non-specific	Intellectual disabilities	Design research; case study	Learner adaptation; learner evaluation; guidance; support of serious games for students with disabilities	Yes
Terras, Boyle, Ramsay, Jarrett (2018)	Non-specific	Intellectual disabilities	Integrative review	Learning Analytics supports personalised learning; support of serious games for students with disabilities	No
Cano, Fernández-Manjón, García-Tejedor (2018)	Non-specific	Intellectual disabilities	Case study	Learning Analytics supports serious games for students with disabilities; Validation of serious game design for students with disabilities	Yes
Konomi et al. (2018)	Non-specific	Elderly people, unskilled adults	Design research	Support of unskilled adults; Digital support (notifications)	No
Cano, Fernández-Manjón, García-Tejedor (2017)	Non-specific	Intellectual disability	Design research	Learning Analytics supports serious games for students with disabilities; validation of design and improve skills	No
Kourakli et al. (2017)	Pre Higher Education	Special educational needs such as dyslexia, dyspraxia, dyscalculia and ADHD	Pilot study	Learning Analytics provides reports that examine children cognitive, motor and academic skills improvement	Yes
Mejia et al. (2017)	Higher Education	Reading disability	Case study; design research	Learning Analytics promotes awareness	Yes
Cano, Fernandez-Manjon, Garcia-Tejedor (2016)	Non-specific	Intellectual disability	Design research	Learning Analytics improves the development of learning games for SWDs	No
Cooper, Ferguson, & Wolff (2016)	Higher Education	Disability (general)	Case study	Learning Analytics identifies dropout and behaviour	Yes
Buzzi, Buzzi, Perrone, Rapisarda, Senette (2016)	Pre Higher Education	disability (Down syndrome)	Design research; pilot study	Learning Analytics helps monitoring students; support of learning; personalisation	Yes

Of the 26 papers in our final corpus, surprisingly, none were published earlier than 2016. The majority of the articles were published recently in 2021 (n = 7) followed by 2020 (n = 5); (Others were published as follows: 2016 (3); 2017 (3); 2018 (4); 2019 (3); 2022 (1) - bearing in mind that 2022 is an incomplete picture). This suggests that research interest is growing in the field and that issues of inclusiveness are re-emerging as of greater importance to institutional stakeholders and educational researchers.

The dominant context in terms of where learning analytics had been applied or studied was in higher education, with half of the studies related to HE (n = 13). Generic contexts (non/specific) of the papers accounted for (n = 5). Studies on schools were (n = 4), adult education (n = 3), and special education (n = 1). One study by Costas-Jauregui et al. (2021) examined both contexts of pre-higher education and higher education.

With respect to the description of inclusiveness of the final literature collection (N.B. some studies share multiple aspects of inclusiveness), a large body of the scientific papers covered cognitive disability (n = 13), followed by other generic types of disability (n = 5). Socially disadvantaged groups accounted for (n = 5). Age and gender-related studies accounted for (n = 2) each, and a single study covered English as a First Language as the primary issue.

The majority of studies had been evidenced (n = 17) by case studies, etc. Methodological approaches varied between reviews, case studies, pilot studies, design research, interviews, surveys, and position papers.

### Major themes identified

Table 4 summarises the main themes identified in the included literature studies and lists the associated papers.

**Table 4:** An overview of themes extracted from the final analysis

Theme	Number of occurrence^1^	Articles
Improvement of LA methods	12	Alonso-Fernández et al. (2019); Bayer et al. (2021); Costas-Jauregui et al. (2021); Foster et al. (2020); Hlosta et al. (2021); Konomi et al. (2018); Mohammadhassan et al. (2021); Niemelä et al. (2020); Riazy et al. (2019); Selwyn (2019); Terras et al. (2018); Williamson et al. (2022)
Increase inclusion	9	Buzzi et al. (2016); Cano et al. (2016); Cano et al. (2018); Chen (2020); Cooper et al. (2016); Konomi et al. (2018); Summers et al. (2021); Tamura et al. (2019); Terras et al. (2018)
Reduce discrimination	6	Bayer et al. (2021); Chen (2020); Costas-Jauregui et al. (2021); Hlosta et al. (2021); Riazy et al. (2019); Williamson et al. (2022)
Support	4	Cooper et al. (2016); Foster et al. (2020); Oyelere et al. (2020); Tamura et al. (2019)
Validate learning design	3	Cano et al. (2016); Cano et al. (2018); Kourakli et al. (2017)
Improvement of learning design	3	Alonso-Fernández et al. (2019); Konomi et al. (2018); Niemelä et al. (2020)
Adaptive/personalised teaching & learning	3	Dietrich et al. (2021); Niemelä et al. (2020); Terras et al. (2018)
^1^ studies overlap in themes		

Other themes included, e.g., the production of frameworks for the LA community and guidance for educational application developers; tracking and profiling; improving accessibility; and specific skills development.

## Discussion

In their review of articles (i.e., those published in the Journal of Learning Analytics, the LAK conference and Web Of Science) looking particularly at learning analytics research relating to students with disabilities, Baek and Aguilar (2022) note that the major themes identified were: detecting struggles, promoting learning, evaluating accessibility, and addressing ethics and privacy concerns. In our broader review, we have taken account of studies which consider other aspects of inclusiveness, such as gender, ethnicity, age, etc.

The research question that we have attempted to address here is, What is known about learning analytics in promoting inclusiveness and supporting students with disabilities? In reviewing the state of learning analytics research in terms of both inclusiveness and students with (a known) disability, our findings have highlighted as a primary focus (a need for) the improvement of learning analytics approaches in this context. We found that learning analytics in covering the topics of inclusiveness and students with disabilities is rather limited. Empirical studies are lacking and we found no study examining the topic prior to 2016.

Of those reported, several studies centred particularly on identifying more effective ways for educational institutions to use learning analytics to support disadvantaged students and others reported on specific tools or games with the aim of using the findings to further improve usefulness and validity. In his short position paper, Selwyn (2019) suggests that learning analytics has been somewhat lacking in this area to date, stating that there is room to consider “how might we ‘think otherwise’ about the application of analytics in higher education”. Further, he suggests that this “would certainly include what are euphemistically referred to as ‘non-traditional’ students, as well as students from non-white, non-binary and other marginalized backgrounds”. This is a view supported by several others. Williamson and Kizilcec (2022) state that we should ask “critical questions about how LADs [learning analytics dashboards] are designed and used, especially considering that many institutions are grappling with issues of diversity, equality, and inclusion.“ (p.260). Chen (2020) agrees, declaring that “learning analytics, a relatively new field of research and practice, has not paid much attention to inclusion and accessibility. The lack of accessibility of tools and information can potentially prevent students with disabilities from enjoying the full benefits of learning analytics.“ (p113). Similarly, Costas-Jauregui et al (2021) suggest that “research has been sparse about how to use learning analytics methods to support inclusive education” (p.3) and that “there is a risk of using learning analytics to legitimise the exclusion of certain students” (p.8). This clearly throws up questions for educational institutions to address further.

More positively, many studies highlight the potential benefits that learning analytics can bring. For example, Summers, Higson and Moores (2021) point out that “The ability to detect the effects of disadvantage on student engagement, despite many efforts of the university to mitigate it, would not be possible without the large amount of data available from learning analytics systems” (p.9) Similarly, in their study exploring the potential of analytics to improve accessibility of e-learning and supporting disabled learners, Cooper, Ferguson and Wolff (2016) say that “Analytics provide another way of approaching the problem of identifying where major accessibility deficits lie.” (p.102). Serious games, typically games developed with a purpose going beyond pure entertainment, are also represented here. Several papers discussed the development and application of serious games to support a whole range of intellectual disabilities. For example, a study by Cano et al. (2016) describes the use of a training game to familiarise adult learners with, for example, Down Syndrome, mild cognitive disability and certain types of Autism Spectrum Disorder, in using the subway. Game Learning Analytics techniques collected and analysed learning data whilst users played the videogame, allowing an evaluation of, eg, time completing tasks, inactivity times and the number of correct/incorrect stations while travelling. Others such as Nguyen, Gardner and Sheridan (2018) and Terras et al. (2018) focus on the need for frameworks and guidance for educational application developers when creating serious games for those with intellectual disabilities.

The importance of adequate learning design was also widely considered. Chen (2020) argues that “interface design can create potential barriers for students with disabilities” (p115) and “designers of the learning analytics interface do not have awareness of the potential barriers and knowledge on how to create accessible visualizations and dashboards” (p115).

Two other major themes were around improving inclusion and reducing discrimination. These are clearly linked, in that we might regard inclusion as aiming to ensure that there are equitable opportunities (to access learning, to achieve successful outcomes, etc) for all, whereas discrimination considers the opposite side of the coin, i.e., the (sub)conscious exclusion of individuals or groups based on their characteristics. For example, Williamson and Kizilcec (2022) discuss the use of learning analytics “to help reduce systemic inequities that give rise to socio-demographic achievement gaps and the underrepresentation of historically disadvantaged groups” (p.261) and “our goal … to indicate places where small intentional changes could actively help dismantle injustices in education.“ (p.269). Riazy and Simbeck (2019) discuss evidence of gender discrimination in their study of predictive analytics stating that “all models predicted below average pass rates for female course participants, where they were higher in reality” (p. 227).

Other themes looked at applications of learning analytics in more ‘traditional’ ways, for example, by proactively tracking students and providing intervention and support. Hlosta et al (2021) looked at the potential benefits of such approaches, but flagged that more work was needed to fully understand how LA might consistently benefit disadvantaged students. They state that there is “growing evidence to suggest that using predictive LA to trigger interventions leads to improved student outcomes in some studies but not in others. This suggests that further fine-grained analysis is needed to understand which of the students may benefit the most from PLA [predictive learning analytics] interventions.“ (p.191). However, the authors did conclude that socio-economically disadvantaged students “are more likely to benefit from PLA systems” (p.194).

In exploring the effectiveness of learning analytics for identifying at-risk students, Foster and Siddle (2020) noted that students from a widening participation background were around 43% more likely to generate a ‘non engagement’ alert. However, the authors guard against focusing solely on demographics, noting that this might be counterproductive. They state that “over ¾ of widening participation students progressed … Whilst there is no dispute that targeting additional resources to help them overcome barriers such as acculturation or help them access financial support or other professional services may be beneficial, using background is inefficient and risks patronising or demotivating students who are coping perfectly well.“ (p2).

Greater use of student data via learning analytics may, however, infringe on their rights to privacy. Such rights are protected by legislation (e.g., the General Data Protection Regulation, GDPR), and so there is a need to ensure the ethical collection, analysis, and use of student data. While supporting disadvantaged students and/or those with disabilities through learning analytics is increasingly researched, the corresponding ethical issues around the use of their data in learning analytics, are not yet consistently considered.

What has emerged from this study is the breadth of issues linked to notions of ‘disability’ and ‘inclusiveness’. Whilst many might consider disability in fairly one-dimensional terms, this review has unearthed studies which incorporate a range of physical, emotional, learning and intellectual disabilities, each needing a tailored approach. Similarly, approaches taken to improve inclusiveness go beyond the simple desire to provide access to opportunities and resources for those who might otherwise be excluded or marginalised. Whilst there is no simple solution, it is heartening to see recognition that learning analytics can and should be put to better uses. As Tsikinas and; Xinogalos. (2021) say in their discussion of the uses of serious games in Special Education schools, “learning analytics can help to address the gap between an increasingly diverse student population and a “one-size-fits-all” approach in education.“ (p117).

### Limitations of this study

We acknowledge that the selection of the two databases, excluding reports and documents other than conference proceedings and journal articles, and restricting the language to English are limitations here. We also acknowledge that there is the possibility of other documents that fit the theme of this paper (i.e., inclusiveness, disability, and disadvantaged students) which may have been yielded by the use of different keywords than those used in this study.

## Conclusions

Although crucial to consider explicitly issues of inclusion or exclusion, we agree also with Hillaire et al., (2016) that implementation of learning analytics applications for disadvantaged students should be done in an inclusive manner to “challenge, motivate, support, and educate not only students with learning disabilities, but their peers (and teachers) too” (Hillaire et al., 2016, p.119). Our systematic review on what is known of learning analytics with regard to broader aspects of inclusiveness and disability highlights that much remains to be done. Although the potential to improve matters is huge, it is not enough simply to search for ways to ameliorate the effects of learning analytics on disadvantaged groups and individuals. Nor is it enough to open the doors of education to those previously denied. Rather, it is the responsibility of society and of educational institutions to actively seek ways to adopt learning analytics and other technological approaches to directly improve their chances of access and of success, and “serve majority and minority groups with the same effectiveness.“ (Bayer et al., 2021, p.71). Selwyn (2019) is right to say that it is time now to think ‘otherwise’ about how we best use learning analytics.

## Footnotes

1 https://www.solaresearch.org/about/what-is-learning-analytics/ (last accessed: March 2022).
